# Dynamics of a Magnetic Polaron in an Antiferromagnet

**DOI:** 10.3390/ma17020469

**Published:** 2024-01-18

**Authors:** Kaijun Shen, Maxim F. Gelin, Kewei Sun, Yang Zhao

**Affiliations:** 1 School of Materials Science and Engineering, Nanyang Technological University, Singapore 639798, Singapore; 2 School of Science, Hangzhou Dianzi University, Hangzhou 310018, China

**Keywords:** variational method, coherent states, Davydov Ansatz, t-J model, hole dynamics, magnetic polarons

## Abstract

The t-J model remains an indispensable construct in high-temperature superconductivity research, bridging the gap between charge dynamics and spin interactions within antiferromagnetic matrices. This study employs the multiple Davydov Ansatz method with thermo-field dynamics to dissect the zero-temperature and finite-temperature behaviors. We uncover the nuanced dependence of hole and spin deviation dynamics on the spin–spin coupling parameter J, revealing a thermally-activated landscape where hole mobilities and spin deviations exhibit a distinct temperature-dependent relationship. This numerically accurate thermal perspective augments our understanding of charge and spin dynamics in an antiferromagnet.

## 1. Introduction

The exploration of charge carriers in doped antiferromagnetic (AFM) layers remains a cornerstone in the realm of quantum many-body physics, a pursuit invigorated by the discovery of high-temperature superconductivity in cuprates [1,2,3,4,5,6,7,8,9,10]. This enigma extends across various two-dimensional (2D) materials, including pnictides [11], organic layers [12], and twisted bilayer graphene [13], where the interplay of hole motion and AFM order emerges as a crucial piece in the puzzle of unconventional superconductivity [14,15]. Recent experimental advances, particularly the use of ultracold atoms in optical lattices [16,17,18,19,20], have rekindled interest in mobile charge carriers in quantum AFM magnets. These cutting-edge experiments offer a near-ideal realization of the Hubbard model, enabling a closer look into the interplay between charge carriers and magnetic order in doped antiferromagnets [21,22,23,24]. The emergence of new geometries, such as bilayers [25] and ladders [26] within these optical lattices, provides further opportunities to enrich our understanding of these complex systems.

The t-J model, which intuitively combines the Heisenberg AFM term with a hole-hopping component, has become a paradigm in our quest to understand charge-spin dynamics in strongly correlated systems. Interactions between charge carriers and magnetic excitations have been shown to significantly influence the transport properties of many-body systems, as evidenced in phenomena such as the Kondo effect, colossal magnetoresistance, and heavy-fermion materials. The 2D Hubbard model epitomizes this interaction, revealing a rich tapestry of dynamics even at the level of a single charge excitation. The creation and dispersion of magnetic polarons within this model underscore the nuanced nature of these interactions [27,28], where out-of-equilibrium characteristics are pivotal in unraveling the mysteries of transport in strongly correlated materials. The model’s predictions, particularly regarding the unusual positioning of quasiparticle band minima and the development of hole pockets, have been verified in angle-resolved photoemission spectroscopy (ARPES) studies [29,30,31,32,33], thereby cementing its role in the narrative of cuprate superconductivity.

Notably, at the crux of the t-J model lies the concept of magnetic polarons [27,28,34,35,36]. The self-consistent Born approximation (SCBA) [29] has been used to describe the equilibrium [28,36,37,38,39] and nonequilibrium properties [40] of these polarons, affirming the t-J model’s prowess in elucidating the dynamics within doped AF layers, including multilayer systems [41,42,43,44,45,46,47]. The t-J model at finite temperatures has also been treated with SCBA [48] and its variants [49,50]. Nonetheless, inter-site correlations are approximated via a mean-field approach in the SCBA, which elides the nuanced dynamical intricacies manifest at femtosecond time scales with an incomplete portrayal of magnon dynamics. However, the quest to elucidate the microscale mechanisms underpinning high-temperature superconductivity [14,15,51], coupled with a discovery that a hole, even when interacting with the spin environment, may sustain quantum coherence at infinitely high temperatures [17], necessitates an expanded theoretical treatment that transcends the confines of mean-field approximations.

In this work, we adopt the multiple Davydov Ansatz (mDA) formalism [52] with the thermo-field dynamics (TFD) method [53,54,55,56] to formulate a computationally accurate approach for elucidating the finite-temperature hole behavior and the spin deviation in the t-J model. Namely, the TFD representation of quantum mechanics combined with the thermal Bogoliubov transformation permits us to treat the finite-temperature dynamics generated by the t-J Hamiltonian via the thermal time-dependent Schrödinger equation, while the variational mDA approach allows us to obtain a numerically accurate solution to this equation without resorting to additional approximations.

In the subsequent sections, we embark on a detailed exploration of the t-J model, delving into its multifaceted aspects and many implications thereof. Section 2 commences with an introduction to the t-J model and the methodologies employed in our study. This is followed by Section 3, which presents an extensive discussion of our results, dissecting the finite-temperature dynamics of a single hole and its surrounding magnons. In Section 3.1, we scrutinize the spatial spread and momentum distribution of the hole at zero temperature, alongside the dynamics of spin deviations at various lattice sites under varying strengths of the spin–spin exchange interaction. The impact of temperature on the hole dynamics and spin-deviation evolution is discussed in Section 3.2. The paper ends in Section 4 with our conclusions.

## 2. t-J Model and Methodologies

The t-J model encapsulates the intricate interplay of charge kinetics and magnon clouds, where the hole is introduced by doping. Within the framework of a slave-fermion representation, the system is characterized by a Hamiltonian that is rigorously defined in prior studies [27,35,57].

(1) $$\begin{array}{c} \hat{H}=\sum_{q}\omega_{q}{\hat{\beta }}_{q}^{†}{\hat{\beta }}_{q}+\frac{tz}{\sqrt{N}}\sum_{kq}{\hat{h}}_{k-q}^{†}{\hat{h}}_{k}\left[\left(u_{q}\gamma_{k-q}+v_{q}\gamma_{k}\right){\hat{\beta }}_{q}^{†}+\left(u_{q}\gamma_{k}+v_{q}\gamma_{k-q}\right){\hat{\beta }}_{-q}\right] \end{array}$$

where ${\hat{h}}_{q}^{†}$ (${\hat{h}}_{q}$) creates (destroys) a hole, ${\hat{\beta }}_{q}^{†}$ (${\hat{\beta }}_{q}$) is the creation (annihilation) operator of a magnon with crystal momentum *q* and energy $\omega_{q}=JzS\sqrt{1-\alpha^{2}\gamma_{q}^{2}}$, *t* is the hopping strength, and

(2) $$\begin{array}{c} u_{q}=\sqrt{\frac{JzS+\omega_{q}}{2\omega_{q}}},\;\;v_{q}=-\mathrm{sgn}\left(\gamma_{q}\right)\sqrt{\frac{JzS-\omega_{q}}{2\omega_{q}}} \end{array}$$

are the coupling coefficients. Herein, *J* denotes the AFM coupling constant corresponding to nearest-neighbor spin interactions, with *z* and *N* representing the coordination number and the total number of lattice sites, respectively. We adopt the spin quantum number S=1/2, set α=1 for the Heisenberg limit indicating the isotropic spin–spin interactions, and the structure factor $\gamma_{q}=\left[\cos\left(q_{x}\right)+\cos\left(q_{y}\right)\right]/2$ presumes a normalized lattice constant.

For the above Hamiltonian (1), the finite-temperature mDA of multiplicity *M* can be written as follows

(3) $$\begin{array}{c} |D_{2}^{M}\left(\tau \right)\rangle =\sum_{Q}^{N}|Q\rangle \sum_{k}^{M}A_{Qk}\left(\tau \right)e^{\sum_{\left\{q\right\}}\left(f_{k\left\{q\right\}}\left(\tau \right)\beta_{\left\{q\right\}}^{†}-H.C.\right)}|0\rangle \end{array}$$

Here, H.C. denotes the Hermitian conjugate, and |0〉 encapsulates the vacuum state for the magnons. |Q〉 indicates a momentum index for the hole state in the first Brillouin zone, N=64, $\left\{q\right\}=q⊕\tilde{q}$, $\tilde{q}$ is the “tilde” momentum-absorbing temperature effects in the TFD–mDA method [53,54,58], and $f_{k\{q\}}\left(t\right)$ is the magnon displacement with momentum **q** and tilde momentum $\tilde{q}$ in the *k*th coherent state. The initial parameters of momentum-space $A_{Qk}$ are obtained after Fourier transforms to ensure that the single hole occupies the center in the site space. The initial elements of $f_{k\{q\}}$ are random numbers of the order $∼10^{-3}$ because small initial spin deviations are essential to jump start the hole motion. The solution of $A_{Qk}$ and $f_{k\{q\}}$ can be found in Appendix A. Then the total Hamiltonian acting in the extended {q} Hilbert space assumes the form [59]

(4) $$\begin{array}{c} \bar{H}=H-\sum_{q}\omega_{q}{\tilde{\beta }}_{q}^{†}{\tilde{\beta }}_{q} \end{array}$$

where ${\tilde{\beta }}_{q}^{†}$ and ${\tilde{\beta }}_{q}$ are the tilde creation and annihilation operators. Having performed thermal Bogoliubov transformation specified by the operator

(5) $$\begin{array}{c} G=G^{†}=-i\sum_{q}\theta_{q}\left(\beta_{q}{\tilde{\beta }}_{q}-\beta_{q}^{†}{\tilde{\beta }}_{q}^{†}\right) \end{array}$$

and following the prescriptions of [59,60], we obtain the final thermo-field dynamics t-J Hamiltonian

(6) $$\begin{array}{c} H_{\theta }=e^{iG}\bar{H}e^{-iG}\\ =\sum_{q}\omega_{q}\left(\beta_{q}^{†}\beta_{q}-{\tilde{\beta }}_{q}^{†}{\tilde{\beta }}_{q}\right)+\frac{tz}{\sqrt{N}}\sum_{kq}h_{k-q}^{†}h_{k}\cosh\left(\theta_{q}\right)[\left(u_{q}\gamma_{k-q}+v_{q}\gamma_{k}\right)\beta_{q}^{†}\\ +\left(u_{q}\gamma_{k}+v_{q}\gamma_{k-q}\right)\beta_{-q}]+\frac{tz}{\sqrt{N}}\sum_{kq}h_{k-q}^{†}h_{k}\sinh\left(\theta_{q}\right)[\left(u_{q}\gamma_{k-q}+v_{q}\gamma_{k}\right){\tilde{\beta }}_{q}\\ +\left(u_{q}\gamma_{k}+v_{q}\gamma_{k-q}\right){\tilde{\beta }}_{-q}^{†}]. \end{array}$$

The influence of temperature is imprinted into ${\bar{H}}_{\theta }$ through the temperature-dependent mixing angles

(7) $$\begin{array}{c} \theta_{q}=\mathrm{arctanh}\left(e^{-\beta_{T}\omega_{q}/2}\right) \end{array}$$

which renormalize hole–magnon coupling coefficients and $\beta_{T}=1/K_{B}T$.

For our simulation, we employ an 8×8 lattice grid, yielding a site count of N=64, with each site maintaining four nearest neighbors (z=4). The lattice’s geometric configuration is defined in an x–y coordinate system with its origin at the center, where the lattice positions are demarcated by the vector **d**. The hole’s temporal evolution in the site space is described by the density matrix $\rho_{h}\left(d,\tau \right)$, which adheres to the normalization condition $\sum_{d}\rho_{h}\left(d,\tau \right)=1$. Simultaneously, the momentum–space distribution of the hole is given by $n_{h}\left(q,\tau \right)$ at $q=(q_{x},q_{y})$, where $\sum_{q}n_{h}\left(q,\tau \right)=1$. Our computations are conducted with an mDA multiplicity of M=40 to ensure the accuracy and convergence of our results. d=|d| and the hole is initially positioned at the lattice center (d=0), from which distances $d=0,\;1,\;\sqrt{2},\;2$ are assigned, respectively, to the initial hole site (IHS), nearest neighbors (NNs), next-nearest neighbors (NNNs), and second-nearest neighbors (SNNs). The hole population $n_{h}\left(q,\tau \right)$ in the momentum space can be written as

(8) $$\begin{array}{c} n_{h}\left(q,\tau \right)=\langle D_{2}^{M}\left(\tau \right)|e^{iG}h_{q}^{†}h_{q}e^{-iG}|D_{2}^{M}\left(\tau \right)\rangle \\ =\sum_{p,u}^{M}A_{qp}^{*}A_{qu}R\left(f_{p}^{*},f_{u}\right) \end{array}$$

The hole population $\rho_{h}\left(d,\tau \right)$ can be expressed as

(9) $$\begin{array}{c} \rho_{h}\left(d,\tau \right)=\langle D_{2}^{M}\left(\tau \right)|e^{iG}h_{d}^{†}h_{d}e^{-iG}|D_{2}^{M}\left(\tau \right)\rangle \\ =\langle D_{2}^{M}\left(\tau \right)|\frac{1}{N}\sum_{q_{1}q_{2}}e^{-i(q_{1}-q_{2})d}h_{q_{1}}^{†}h_{q_{2}}|D_{2}^{M}\left(\tau \right)\rangle \\ =\frac{1}{N}\sum_{q_{1}q_{2}}e^{-i(q_{1}-q_{2})d}\sum_{p,u}^{M}A_{q_{1}p}^{*}A_{q_{2}u}R\left(f_{p}^{*},f_{u}\right) \end{array}$$

where the Debye–Waller factor $R(f_{p}^{*},f_{u})$ is written as

(10) $$\begin{array}{c} R\left(f_{p}^{*},f_{u}\right)=\exp\left[\sum_{l}f_{pl}^{*}\left(\tau \right)f_{ul}\left(\tau \right)+\sum_{l}{\tilde{f}}_{pl}^{*}\left(\tau \right){\tilde{f}}_{ul}\left(\tau \right)\right] \end{array}$$

The spin deviations ΔS(d,τ) [61], monotonically related to the magnon population [62], represent the departure from a perfectly ordered AFM state due to the presence of a hole that disrupts the local spin alignment. A higher level of spin deviations from the ordered state indicates an increase in the magnon population. ΔS(d,τ) at each site can be defined as $\Delta S\left(d,\tau \right)=\langle D_{2}^{M}\left(\tau \right)|e^{iG}b_{d}^{†}b_{d}e^{-iG}|D_{2}^{M}\left(\tau \right)\rangle$, whose derivation can be found in the Appendix. The average spin deviation is $\Delta \bar{S}\left(\tau \right)=\sum_{d}\Delta S\left(d,\tau \right)/N$.

## 3. Results and Discussion

### 3.1. Nonequilibrium Quantum Dynamics of a Single Hole

#### 3.1.1. Spatial Spread of a Single Hole

**Figure 1:**
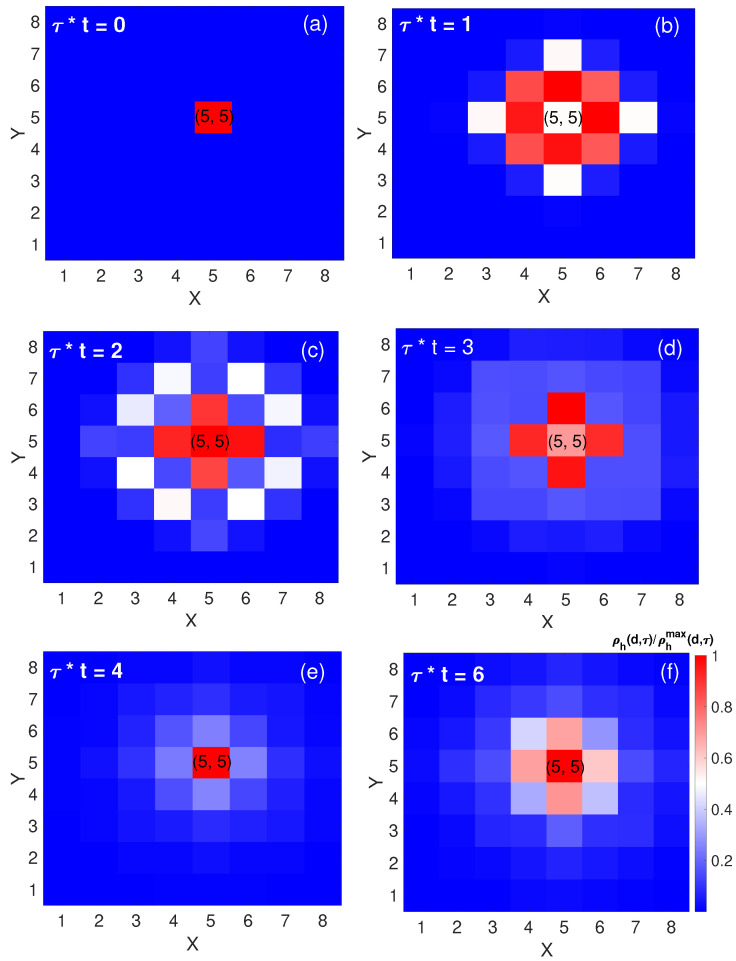
Evolution of single-hole dynamics in an AFM lattice. Panels (**a**–**f**) depict the evolution of hole density, $\rho_{h}\left(d,\tau \right)$, over various times under the condition J=0.233t. τ∗t is used as a dimensionless parameter characterizing the time evolution of the system.

In Figure 1a–f the hole density is plotted to represent its spatial spread, where the hole diffuses along the four NNs within a short time, in agreement with laboratory observation [19]. The simulated picture of hole motion is consistent with the measured probability density of the hole [17] under infinitely strong on-site repulsion, proving the hopping term in t-J model plays a dominant role at a short time. Prior to the temporal marker τ=2/t, the hole exhibits a pronounced expansion towards its NNs. In contrast, at subsequent intervals τ=3/t and τ=4/t, there is a discernible contraction in the spatial extent of the hole distribution, indicative of a reduction in hole population at both NNs and NNNs. Notably, at τ=6/t, a resurgence in the hole’s spatial distribution appears, extending once more towards the NNs. This dynamical fluctuation in hole population is consistent with the interplay of interfering string excitations [40]. As the hole hops from one site to another, it leaves behind a “string” of disturbed spin states, i.e., a trail of misaligned spins. Since the hole can follow multiple paths in the lattice, the string of disturbed spins can have different configurations based on the path taken by the hole. All these different paths and string configurations can coexist in a superposition state. When the hole retraces its steps or takes a different path that crosses or interacts with its previous trail, the string states can interfere with each other.

#### 3.1.2. Population Dynamics Comparison with SCBA Method and Experimental Data

The mDA scheme, recognized for its efficacy in variational many-body quantum mechanics, employs Gaussian state representations and has been adeptly applied to a diverse spectrum of quantum phenomena. These range from the investigation of Landau–Zener transitions [63] and the dynamics of cavity polaritons [64], to the mechanisms underlying excitonic energy transfer in photosynthetic complexes [65], as well as to the interrogation of ultrafast spectroscopic processes at conical intersections [66].

**Figure 2:**
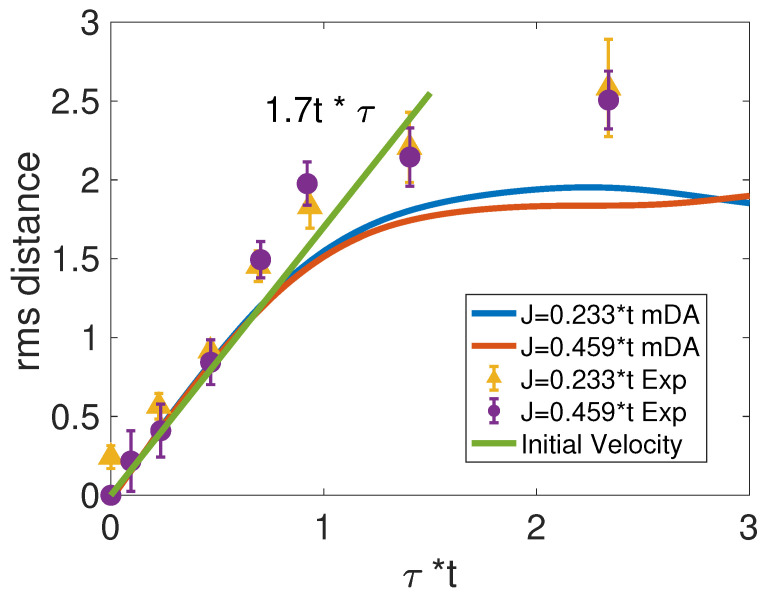
The rms distance comparison between mDA method and experimental measurements [23] under two different J/t ratios. The green line represents the initial velocity.

**Figure 3:**
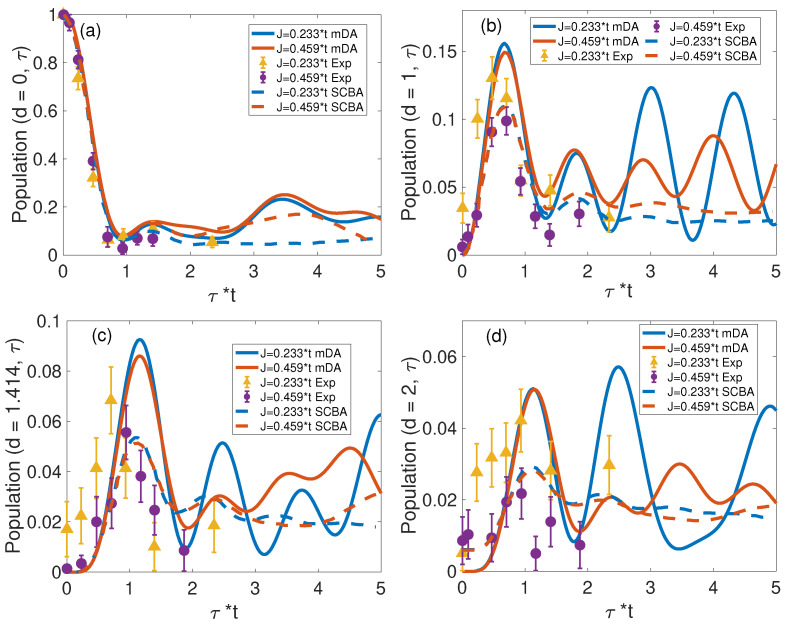
Hole population calculated by the mDA is compared with that by SCBA method [40], and the measured data [23]. Panels (**a**–**d**) display the hole population in the IHS, NN, NNN, and SNN areas, respectively.

Calculated from the hole density, the root-mean-square (rms) distance $d_{rms}\left(\tau \right)={\left[\sum_{d}d^{2}\rho_{h}\left(d,\tau \right)\right]}^{1/2}$ is compared with measurements [23] in Figure 2 for J/t=0.233 and J/t=0.459. Nielsen et al. [40] first found the period of τ<1/t to be quantum walk, indicating the initial ballistic motion of the itinerant hole. The initial velocity $v=\partial_{\tau }d_{rms}$ from the mDA method is 1.7t, in agreement with measurements [23]. For τ>1/t, the hole velocity decreases significantly thanks to magnon dressing of the hole. This leads to the coherent oscillations of the hole population as shown in Figure 3 [39,67].

In Figure 3, $\rho_{h}\left(d,\tau \right)$ computed by the mDA is compared with that computed by the SCBA method [40] and the measured data [23]. Overall, the agreement between our mDA results and measurements is especially good for d=0 and d=1, as shown in Figure 3a,b, respectively. Yet, there exist substantial differences: at odds with measurements and mDA predictions, SCBA underestimates amplitudes of the first population recurrence and does not reproduce the difference of their amplitudes for the two values of the spin coupling *J* in Figure 3b. This deviation can be attributed to the crude treatment of inter-site correlations in mean-field-based SCBA. We thus conclude that the nonperturbative mDA methodology is superior to SCBA in predicting the short-time magnon population dynamics. Comparisons with measurements at larger distances, however, are impeded by diminishing signal–to–noise ratios, while the finite-size effect in our mDA computation seems to accentuate the revival of oscillations of the NNN and SNN populations.

#### 3.1.3. Site-Dependent Hole Dynamics across Diverse Spin–Spin Interaction Regimes

**Figure 4:**
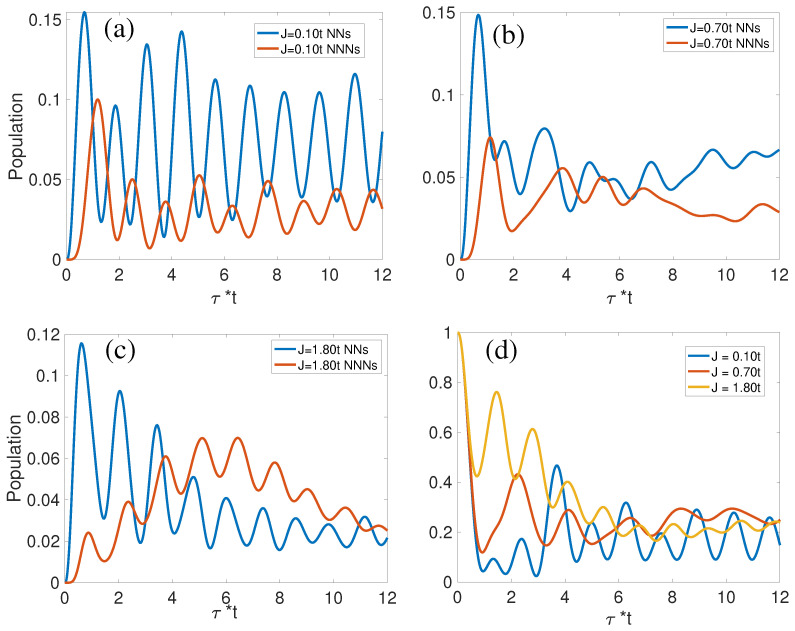
Comparative analysis of hole populations at NNs and NNNs. Panels (**a**–**c**) display the variations in hole populations for a range of *J* values, while panel (**d**) provides a focused view of the hole population at the IHS for varying J/t ratios.

The hole dynamics in Figure 4a–c reveal competition between NNs and NNNs with the nuanced influence of the spin–spin exchange interaction. In scenarios characterized by a subdued spin–spin exchange interaction, as shown in Figure 4a, the hole exhibits a propensity to disperse predominantly through NNs, maintaining a consistently higher population at NNs (compared to NNNs) for the majority of the observed time frame. However, with an intensification of *J*, the hole’s population at NNNs not only rivals that at NNs, as depicted in Figure 4b, but frequently surpasses it over extended periods in Figure 4c. This is due to strong spin–spin interactions maintaining the AFM order. The hole wave function spreads out to various lattice sites. Over time, the probability distribution of the hole position evolves to favor sites where the spin disruption is minimized. This can lead to a higher probability of finding the hole at NNNs, where the paths it has taken result in a lower overall energy cost.

Figure 4d delineates the implications of varying *J* values on hole dynamics specifically at the IHS. A heightened *J* value imposes constraints on the hole’s mobility in the initial stages, attributable to the effect of spin–spin interactions upholding a rigid AFM milieu. Conversely, in a lattice where the spin–spin coupling *J* is diminished, the hole is more inclined to traverse greater distances from its original location. Intriguingly, a lower *J* value concurrently signifies a more intricate dressing of the hole by spin waves, culminating in its final polaron state. For minuscule *J* values, this intricate dressing mechanism overpowers the reduced energy penalty associated with ∼J in the initial phase, effectively anchoring the hole near its initial position and compelling a return. Consequently, in the small *J* regime, a marked decrease in hole population is found at the IHS before τ=1/t, followed by a distinct resurgence.

#### 3.1.4. Momentum–Space Profile of a Solitary Hole

**Figure 5:**
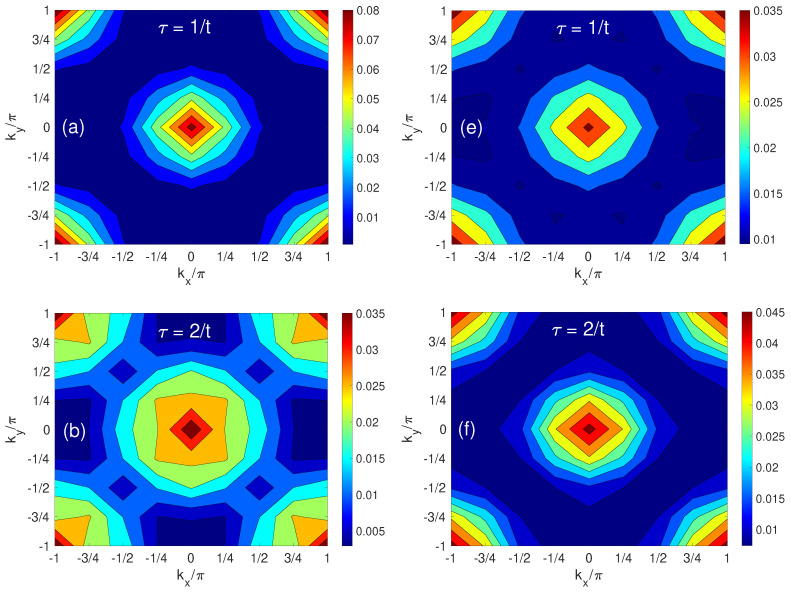

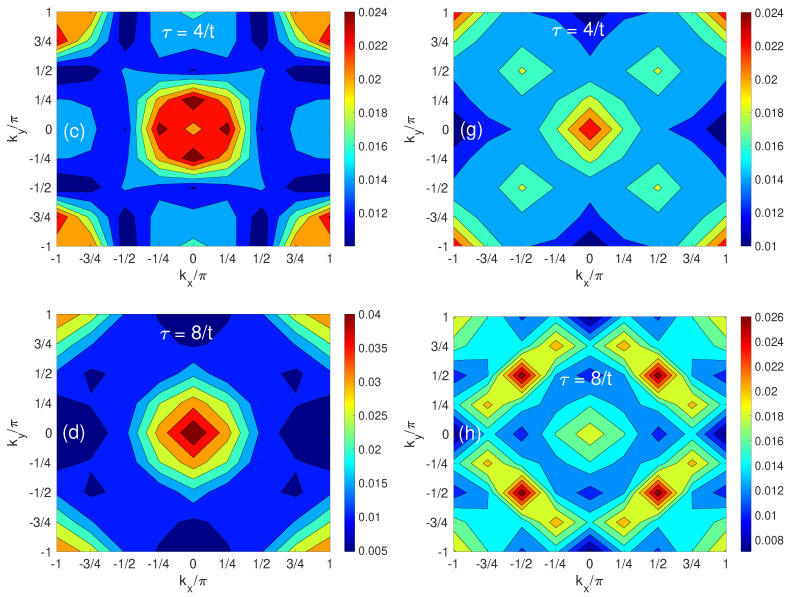
Momentum−space distribution of the hole density in the first Brillouin zone. Contour plots in this figure reveal the hole density $n_{h}\left(q,\tau \right)$ within the first Brillouin zone. The left column (**a**–**d**) illustrates the scenario for a smaller J/t ratio of 0.10 at time τ=1/t, 2/t, 4/t and 8/t, respectively; while the right column (**e**–**h**) explores the dynamics under a larger J/t ratio of 1.8 at those times.

**Figure 6:**
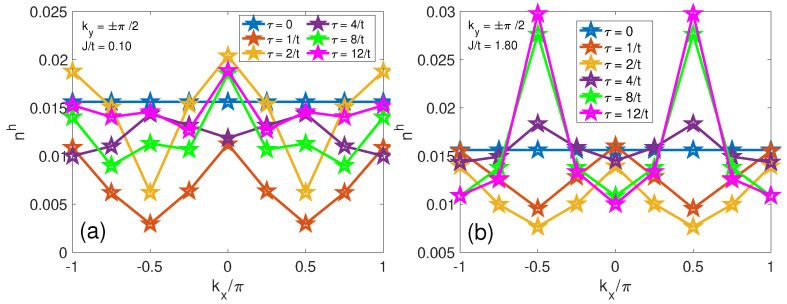
Temporal and momentum−space analysis of the hole density at $k_{y}=\pm \pi /2$. Panel (**a**) focuses on the hole density $n_{h}\left(q,\tau \right)$ at moments when $k_{y}=\pm \pi /2$ for a smaller J/t ratio of 0.10, whereas panel (**b**) corresponds to a larger J/t ratio of 1.80.

Figure 5 presents the evolution of the hole population in the momentum space, delineating the contrast between a regime of small spin–spin interactions (J/t=0.10), shown in panels (a–d), and one characterized by substantial *J* (J/t=1.8), as illustrated in panels (e–h). At τ=0, the momentum distribution spans uniformly across the entire first Brillouin zone, reflecting the initial localization of the hole at a singular lattice site in the site space. Figure 6 presents the momentum–space distribution of a hole at $k_{y}=\pm \pi /2$ under two different spin–spin interaction strengths, *J*. In Figure 6a,b (corresponding to $k_{y}=\pm \pi /2$), the population oscillation indicates entangled string excitations [40].

For small *J*, the spin–spin interaction is weaker, leading to a more delocalized hole pattern. The AFM background is less disrupted by the hole’s motion, allowing the hole to propagate more freely across the lattice. This freedom is manifested in the momentum distribution with maximum hole population located around (0,0) and (±π,±π) as shown in Figure 5a–d, corresponding to the free-particle-like dispersion of holes in a less constrained spin background [27].

From a low *J* to a large *J*, a notable shift in the hole population is found at τ=4/t,8/t and 12/t, particularly at momentum positions $q^{0}=\left(\pm \pi /2,\pm \pi /2\right)$, as evidenced in Figure 5g,h and Figure 6b. This redistribution crescendos to the emergence of four quasiparticle hole “pockets” that correspond to the minimal energy points on the hole (viewed as a spinless fermion) Fermi surface [48,68,69]. For strong spin–spin coupling, the system is close to the Heisenberg limit, where the hole motion is strongly influenced by the spin background, leading to the formation of a magnetic polaron. The corresponding dispersion of the quasiparticle has the energy minima at $q^{0}$ as revealed by the variational methods [57] and SCBA finite-size calculations [27,28,35,36], with the highest hole density found at $q^{0}$. The four peaks of $n_{h}\left(q,\tau \right)$ are also included in the one-hole ground state Ansatz of Chen and coworkers [69] and their calculated quasiparticle spectral weight.

**Figure 7:**
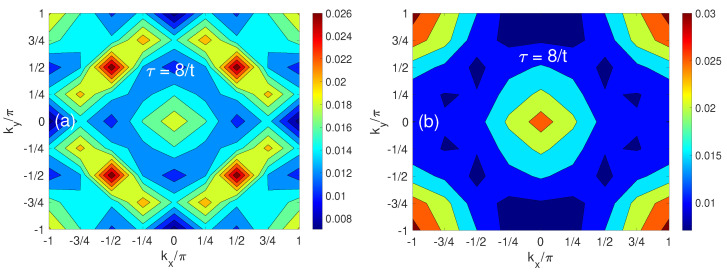
Comparison between T=0 (**a**) and T=1.25J (**b**) for J/t=1.80 at τ=8/t.

The temperature effects on the momentum distribution of the hole are exhibited in Figure 7, which compares the cases of T=0 (a) and T=1.25J (b) for J/t=1.80 at τ=8/t. A significant change is that four pockets at (±π/2,±π/2) are washed out at a high temperature, where the hole population of four pockets decreases dramatically. This effect is similar to the findings of Plakida et al. [48], which is attributed to the temperature shift of the quasiparticle (QP) peak position in the QP spectrum. At high temperatures all QP peaks appear above the chemical potential, leading to positive QP peak positions [48]. Hence, the Fermi factor renders a substantial reduction in the occupancy weight (relative to the low-temperature value of the QP spectral weight), resulting in the vanishing quasihole pockets.

### 3.2. Analysis of Hole Dynamics and Spin Deviations at Finite Temperatures

#### 3.2.1. Zero-Temperature Spin Deviations

**Figure 8:**
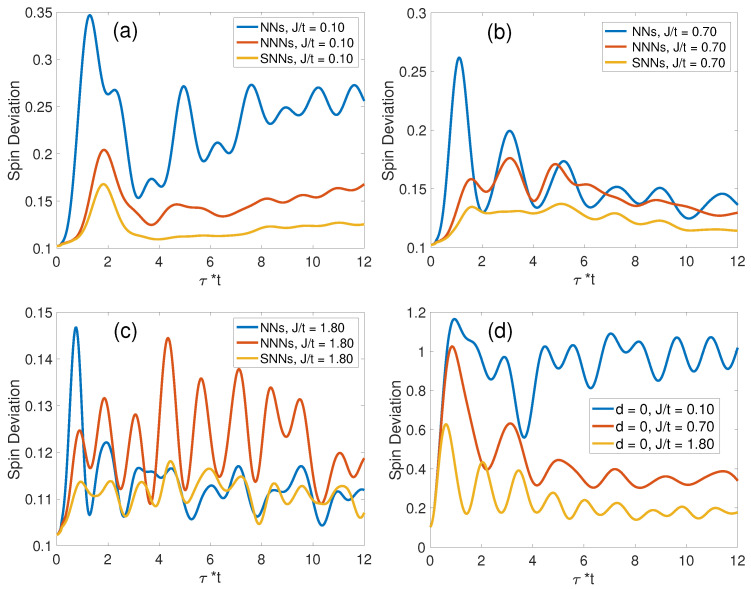
Dynamics of spin deviation over time. Panels (**a**–**c**) explore the spin deviation dynamics at different interaction strengths, while panel (**d**) specifically examines the spin deviations at the IHS across a spectrum of J/t ratios.

Figure 8 illustrates the dynamics of spin deviations for NNs and NNNs, reflecting a competitive behavior found in the distribution of hole populations at these respective sites. For large *J*, as illustrated in Figure 8a–c, there is an enhanced oscillatory behavior in spin deviations at both NNs, NNNs, and SNNs. These oscillations, marked by their periodicity of τ=1.3/t in Figure 8c, are indicative of heightened magnon energies elicited by the hole’s motion. For J/t=1.80, there is a consequential suppression of magnon generation at NNs, ultimately resulting in their spin deviations subsiding to levels below those of NNNs, as captured in Figure 8c. Conversely, under a regime of diminished *J*, where hole mobility is comparatively higher, there is a notable reduction in hole occupancy at the IHS, as demonstrated in Figure 4d. This increased mobility precipitates a marked spin deviation at the IHS, as explicitly presented in Figure 8d. The dynamics encapsulated in these observations are pivotal for understanding the underlying spin–charge interactions and their manifestations in the t-J model.

#### 3.2.2. Finite-Temperature Hole Population Dynamics

**Figure 9:**
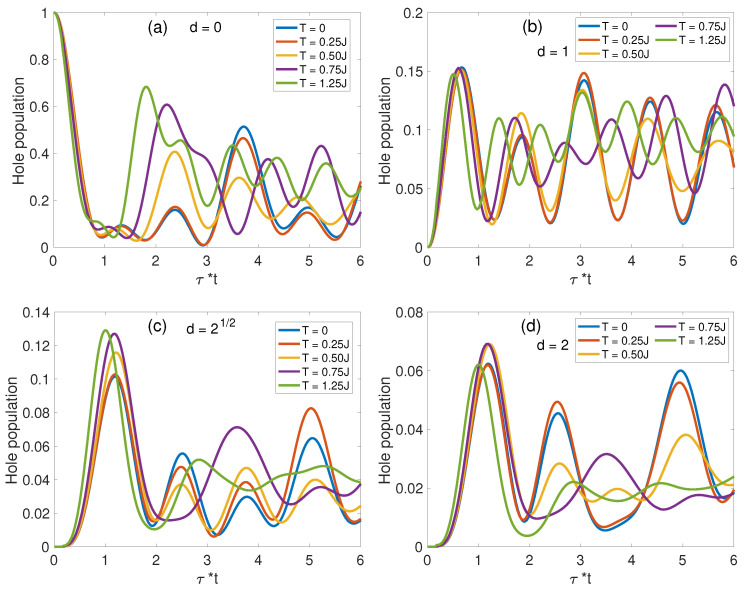
Temperature-dependent hole population at various sites for J/t=0.2. Panels (**a**–**d**) display $\rho_{h}\left(d,\tau \right)$ in the IHS, NN, NNN, and SNN areas, respectively.

Figure 9 provides a detailed temporal evolution of hole densities $\rho_{h}\left(d,\tau \right)$ across a range of temperatures, where J/t=0.2 is a typical value for high-$T_{c}$ cuprate superconductors [15]. From a broad perspective, hole densities are characterized by significant temporal fluctuations, which persist from absolute zero temperature—aligning with tensor-network predictions [70]—to higher temperatures. Notably, as the temperature is escalated, both the amplitude and periods of these oscillations in the IHS (a) and NNs (b) regions expand, reflecting temperature-dependent enhancements in hole–hole interactions mediated by an increased ensemble of thermally excited magnon states that dictate the interaction strength. This effect can be modeled in the paradigm of an effective Rabi model, where the intensified interaction strength leads to a greater Rabi oscillation frequency, which governs the dynamics of hole population. Conversely, for the NNNs (c) and SNNs (d) regions, the hole population displays less discernible patterns in their thermal evolution of amplitudes and frequencies due to the complex interplay of multi-range scattering processes that result in quantum dephasing. Specifically, the IHS hole densities in Figure 9a showcase marked recurrences whose magnitude (and inversely, duration) scales with temperature. The dynamics of NNs hole populations (b) follow a similar behavior as the IHS hole densities, whereas the NNNs (c) and SNNs (d) hole densities demonstrate an irreversible quenching with increased temperature.

#### 3.2.3. Finite-Temperature Spin Deviation Evolutions

**Figure 10:**
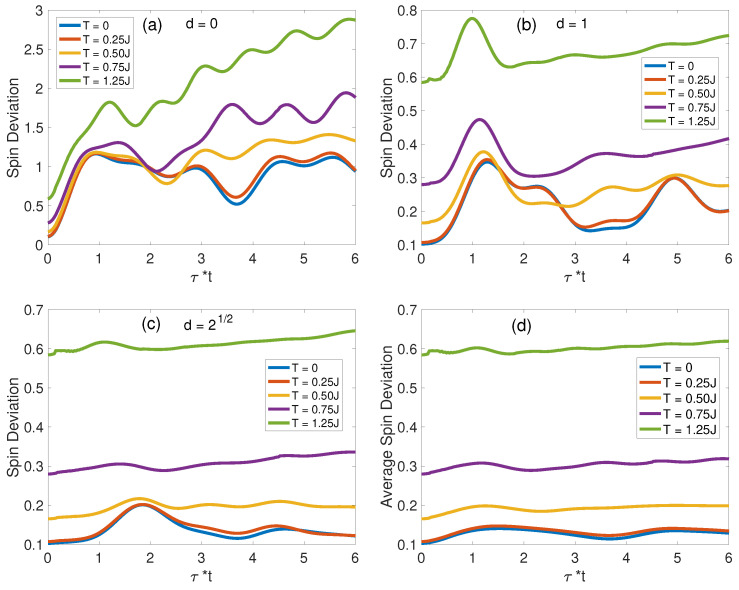
Temperature-dependent spin deviations in the IHS, NN, and NNN areas for J/t=0.2. Panels (**a**–**c**) display the variations in spin deviations for a range of *T* values, while panel (**d**) provides $\Delta \bar{S}\left(\tau \right)$ for varying T/J ratios.

The behavior of spin deviations at J/t=0.2, as depicted in Figure 10a–c, exhibits two salient characteristics. Initially, ΔS(d,τ) displays an escalation as a function of temperature, indicative of the thermally induced activation of magnon states. Subsequently, with the exception of ΔS(0,τ), the spin deviations swiftly converge to equilibrium distributions, a phenomenon that is particularly pronounced at elevated temperatures. The mean spin deviation $\Delta \bar{S}\left(\tau \right)$ increases monotonically with *T* in Figure 10d. This trend is indicative of thermally induced frustration in spin alignment, which agrees with the observed reduction in saturation magnetization at higher thermal states [71,72].

## 4. Conclusions

In this work, we employ the mDA–TFD method to simulate the evolution of hole populations (both in site and momentum space) and spin deviations at finite temperatures. It is found that at zero temperature, the hole population in site space can be significantly affected by the spin–spin interaction, where a large *J* dictates the localization of hole populations. In addition, the spin–spin interaction determines the peak positions of the hole population in the momentum space as well as the spin deviation. Furthermore, the thermal field has substantial impacts on the hole population dynamics. High temperatures enhance temporal oscillations in $\rho_{h}\left(d,\tau \right)$ in the IHS and NN areas, because of temperature-dependent enhancements in hole–hole interactions mediated by an increased ensemble of thermally excited magnon states, but quench those in the NNN and SNN areas. This is an indication of the complex multi-range scattering dynamic and quantum dephasing. More magnons can be excited at a higher temperature, revealing intriguing possibilities for thermally manipulating magnon excitations.

Our mDA–TFD method serves as a robust tool for analyzing the quantum dynamics of impurities in lattice structures. The mDA–TFD methodology developed here can be readily extended to the boson–holon model including an additional hole–phonon coupling Hamiltonian [73]. It can be applied to the simulation of steady-state angular-resolved photoemission spectra [74,75] and femtosecond terahertz pump–probe signals. This framework also has the potential to extend its application to an analysis of magnon polaritons [76], AFM bilayers [77], and nonequilibrium dynamics of multiple holes [78] in strongly interacting lattice models. One direct application of our method is that the calculated population can be compared to the measured density-resolved dynamics of a single hole from a 2D Hubbard insulator, shedding light on the interference phenomenon. Additionally, the rms distance is easy to calculate, contributing to the explanation of long-time hole delocalization in the experiment [23]. Furthermore, the spin dynamics, such as spin correlations, can be calculated by mDA methodology as well, which is expected to help understand the reversal and recovery of AFM correlations observed recently in cold-atom experiments [22,23]. Looking forward, the mDA–TFD can be utilized to investigate the dynamics of spin-lattice polaron (SLP) formation [79] with a single hole, uncovering more details of the relaxation mechanism, which includes the relaxation of kinetic energy for SLP by emitting local spin and phonon excitations in the first stage and energy transfer between phonon and spin degrees of freedom in the second stage. Such analyses could illuminate the interplay between holes, phonons, and magnons, potentially shedding light on fascinating phenomena such as stripe phases, and d-wave superconductivity [14,15,16,17,18,19,20]. 

## Data Availability

The data presented in this study are available on request from the corresponding author.

## Abbreviations

The following abbreviations are used in this manuscript:

AFM	Antiferromagnetic
2D	Two-dimensional
ARPES	Angle-resolved photoemission spectroscopy
SCBA	Self-consistent Born approximation
mDA	Multiple Davydov Ansatz
TFD	Thermo-field dynamics
NNs	Nearest neighbors
NNNs	Next-nearest neighbors
SNNs	Second-nearest neighbors
SLP	Spin-lattice polaron

## Appendix A. Derivation of Hole–Magnon Dynamics in the t-J Model

The time-dependent variational parameters, $A_{Qk}\left(\tau \right)$ and $f_{kq}\left(\tau \right)$ are determined via the variational principle [52]

(A1) $$\begin{array}{c} \frac{d}{d\tau }\frac{\partial L}{\partial {\dot{\xi }}_{j}^{*}}-\frac{\partial L}{\partial \xi_{j}^{*}}=0, \end{array}$$

where the Lagrangian L is given by

(A2) $$\begin{array}{ccc} L & = & \frac{i}{2}\left[\langle D_{2}^{M}\left(\tau \right)|\frac{\vec{\partial }}{\partial \tau }|D_{2}^{M}\left(\tau \right)\rangle -\langle D_{2}^{M}\left(\tau \right)|\frac{\overset{\leftarrow }{\partial }}{\partial \tau }|D_{2}^{M}\left(\tau \right)\rangle \right]-\langle D_{2}^{M}\left(\tau \right)|H_{\theta }|D_{2}^{M}\left(\tau \right)\rangle . \end{array}$$

The Hamiltonian in the multi-$D_{2}$ Ansatz is defined as

(A3) $$\begin{array}{c} L_{H_{\theta }}=\langle D_{2}^{M}\left(\tau \right)|H_{\theta }|D_{2}^{M}\left(\tau \right)\rangle =\sum_{Q}\sum_{p}^{M}\sum_{u}^{M}A_{Qp}^{*}A_{Qu}\sum_{q}\omega_{q}\left(f_{pq}^{*}f_{uq}-{\tilde{f}}_{pq}^{*}{\tilde{f}}_{uq}\right)R\left(f_{p}^{*},f_{u}\right)\\ +\frac{tz}{\sqrt{N}}\sum_{kq}\sum_{p}^{M}\sum_{u}^{M}A_{\left(k-q\right)p}^{*}A_{\left(k\right)u}[\cosh\left(\theta_{q}\right)\left(u_{q}\gamma_{k-q}+v_{q}\gamma_{k}\right)f_{pq}^{*}\\ +\sinh\left(\theta_{q}\right)\left(u_{q}\gamma_{k-q}+v_{q}\gamma_{k}\right){\tilde{f}}_{uq}]R\left(f_{p}^{*},f_{u}\right)\\ +\frac{tz}{\sqrt{N}}\sum_{kq}\sum_{p}^{M}\sum_{u}^{M}A_{\left(k+q\right)p}^{*}A_{\left(k\right)u}[\cosh\left(\theta_{q}\right)\left(u_{q}\gamma_{k}+v_{q}\gamma_{k+q}\right)f_{uq}\\ +\sinh\left(\theta_{q}\right)\left(u_{q}\gamma_{k}+v_{q}\gamma_{k+q}\right){\tilde{f}}_{pq}^{*}]R\left(f_{p}^{*},f_{u}\right) \end{array}$$

Thus the equations of motion for $A_{Qu}$ assume the form

(A4) $$\begin{array}{c} i\sum_{Q}\sum_{u}^{M}\left[{\dot{A}}_{Qu}+A_{Qu}\sum_{l}f_{pl}^{*}{\dot{f}}_{ul}+A_{Qu}\sum_{l}{\tilde{f}}_{pl}^{*}{\tilde{\dot{f}}}_{ul}\right]R\left(f_{p}^{*},f_{u}\right)\\ =\sum_{u}^{M}A_{Qu}\sum_{q}\omega_{q}\left(f_{pq}^{*}f_{uq}-{\tilde{f}}_{pq}^{*}{\tilde{f}}_{uq}\right)R\left(f_{p}^{*},f_{u}\right)\\ +\frac{tz}{\sqrt{N}}\sum_{kq,k-q=Q}\sum_{u}^{M}A_{\left(k\right)u}\left[\cosh\left(\theta_{q}\right)\left(u_{q}\gamma_{Q}+v_{q}\gamma_{k}\right)f_{pq}^{*}+\sinh\left(\theta_{q}\right)\left(u_{q}\gamma_{Q}+v_{q}\gamma_{k}\right){\tilde{f}}_{uq}\right]R\left(f_{p}^{*},f_{u}\right)\\ +\frac{tz}{\sqrt{N}}\sum_{kq,k+q=Q}\sum_{u}^{M}A_{\left(k\right)u}\left[\cosh\left(\theta_{q}\right)\left(u_{q}\gamma_{k}+v_{q}\gamma_{Q}\right)f_{uq}+\sinh\left(\theta_{q}\right)\left(u_{q}\gamma_{k}+v_{q}\gamma_{Q}\right){\tilde{f}}_{pq}^{*}\right]R\left(f_{p}^{*},f_{u}\right) \end{array}$$

Similarly, the equations of motion for $f_{ul}$ and ${\tilde{f}}_{ul}$ are given by the formulas

(A5) $$\begin{array}{c} i\sum_{Q}\sum_{u}^{M}A_{Qp}^{*}A_{Qu}{\dot{f}}_{ul}R\left(f_{p}^{*},f_{u}\right)+i\sum_{Q}\sum_{u}^{M}\left[A_{Qp}^{*}{\dot{A}}_{Qu}+A_{Qp}^{*}A_{Qu}\left(\sum_{l}f_{pl}^{*}{\dot{f}}_{ul}+\sum_{l}{\tilde{f}}_{pl}^{*}{\tilde{\dot{f}}}_{ul}\right)\right]R\left(f_{p}^{*},f_{u}\right)f_{ul}\\ =\sum_{Q}\sum_{u}^{M}A_{Qp}^{*}A_{Qu}\sum_{q}\omega_{q}\left(f_{pq}^{*}f_{uq}-{\tilde{f}}_{pq}^{*}{\tilde{f}}_{uq}\right)R\left(f_{p}^{*},f_{u}\right)f_{ul}+\sum_{m}\sum_{u}^{M}A_{Qp}^{*}A_{Qu}\omega_{l}f_{ul}R\left(f_{p}^{*},f_{u}\right)\\ +\frac{tz}{\sqrt{N}}\sum_{kq}\sum_{u}^{M}A_{\left(k-q\right)p}^{*}A_{\left(k\right)u}\left[\cosh\left(\theta_{q}\right)\left(u_{q}\gamma_{k-q}+v_{q}\gamma_{k}\right)f_{pq}^{*}+\sinh\left(\theta_{q}\right)\left(u_{q}\gamma_{k-q}+v_{q}\gamma_{k}\right){\tilde{f}}_{uq}\right]R\left(f_{p}^{*},f_{u}\right)f_{ul}\\ +\frac{tz}{\sqrt{N}}\sum_{kq}\sum_{u}^{M}A_{\left(k-q\right)p}^{*}A_{\left(k\right)u}\left[\cosh\left(\theta_{q}\right)\left(u_{q}\gamma_{k}+v_{q}\gamma_{k+q}\right)f_{uq}+\sinh\left(\theta_{q}\right)\left(u_{q}\gamma_{k}+v_{q}\gamma_{k+q}\right){\tilde{f}}_{pq}^{*}\right]R\left(f_{p}^{*},f_{u}\right)f_{ul}\\ +\frac{tz}{\sqrt{N}}\sum_{k}\sum_{u}^{M}A_{\left(k-l\right)p}^{*}A_{\left(k\right)u}[\cosh\left(\theta_{l}\right)\left(u_{l}\gamma_{k-l}+v_{l}\gamma_{k}\right)R\left(f_{p}^{*},f_{u}\right) \end{array}$$

and

(A6) $$\begin{array}{c} i\sum_{Q}\sum_{u}^{M}A_{Qp}^{*}A_{Qu}{\tilde{\dot{f}}}_{ul}R\left(f_{p}^{*},f_{u}\right)+i\sum_{Q}\sum_{u}^{M}\left[A_{Qp}^{*}{\dot{A}}_{Qu}+A_{Qp}^{*}A_{Qu}\left(\sum_{l}f_{pl}^{*}{\dot{f}}_{ul}+\sum_{l}{\tilde{f}}_{pl}^{*}{\tilde{\dot{f}}}_{ul}\right)\right]R\left(f_{p}^{*},f_{u}\right){\tilde{f}}_{ul}\\ =\sum_{Q}\sum_{u}^{M}A_{Qp}^{*}A_{Qu}\sum_{q}\omega_{q}\left(f_{pq}^{*}f_{uq}-{\tilde{f}}_{pq}^{*}{\tilde{f}}_{uq}\right)R\left(f_{p}^{*},f_{u}\right){\tilde{f}}_{ul}-\sum_{Q}\sum_{u}^{M}A_{Qp}^{*}A_{Qu}\omega_{l}{\tilde{f}}_{ul}R\left(f_{p}^{*},f_{u}\right)\\ +\frac{tz}{\sqrt{N}}\sum_{kq}\sum_{u}^{M}A_{\left(k-q\right)p}^{*}A_{\left(k\right)u}\left[\cosh\left(\theta_{q}\right)\left(u_{q}\gamma_{k-q}+v_{q}\gamma_{k}\right)f_{pq}^{*}+\sinh\left(\theta_{q}\right)\left(u_{q}\gamma_{k-q}+v_{q}\gamma_{k}\right){\tilde{f}}_{uq}\right]R\left(f_{p}^{*},f_{u}\right){\tilde{f}}_{ul}\\ +\frac{tz}{\sqrt{N}}\sum_{kq}\sum_{u}^{M}A_{\left(k+q\right)p}^{*}A_{\left(k\right)u}\left[\cosh\left(\theta_{q}\right)\left(u_{q}\gamma_{k}+v_{q}\gamma_{k+q}\right)f_{uq}+\sinh\left(\theta_{q}\right)\left(u_{q}\gamma_{k}+v_{q}\gamma_{k+q}\right){\tilde{f}}_{pq}^{*}\right]R\left(f_{p}^{*},f_{u}\right){\tilde{f}}_{ul}\\ +\frac{tz}{\sqrt{N}}\sum_{k}\sum_{u}^{M}A_{\left(k+l\right)p}^{*}A_{\left(k\right)u}[\sinh\left(\theta_{l}\right)\left(u_{l}\gamma_{k}+v_{l}\gamma_{k+l}\right)R\left(f_{p}^{*},f_{u}\right) \end{array}$$

ΔS(d,τ) at each site can be evaluated as

(A7) $$\begin{array}{c} \Delta S\left(d,\tau \right)=\langle D_{2}^{M}\left(\tau \right)|e^{iG}b_{d}^{†}b_{d}e^{-iG}|D_{2}^{M}\left(\tau \right)\rangle \\ =\langle D_{2}^{M}\left(\tau \right)|\frac{1}{N}\sum_{q_{1}q_{2}}e^{-i(q_{1}-q_{2})d}e^{iG}\left(u_{q_{1}}\beta_{q_{1}}^{†}+v_{q_{1}}\beta_{-q_{1}}\right)\left(u_{q_{2}}\beta_{q_{2}}+v_{q_{2}}\beta_{-q_{2}}^{†}\right)e^{-iG}|D_{2}^{M}\left(\tau \right)\rangle \\ =\frac{1}{N}\sum_{q_{1}q_{2}}e^{-i(q_{1}-q_{2})d}\sum_{Q}\sum_{p,u}^{M}A_{Qp}^{*}A_{Qu}[f_{p(q_{1})}^{*}\left(\tau \right)f_{u(q_{2})}\left(\tau \right)\cosh\left(\theta_{q1}\right)\cosh\left(\theta_{q2}\right)\\ +f_{p(q_{1})}^{*}\left(\tau \right){\tilde{f}}_{p(q_{2})}^{*}\left(\tau \right)\cosh\left(\theta_{q1}\right)\sinh\left(\theta_{q2}\right)+{\tilde{f}}_{u(q_{1})}\left(\tau \right)f_{u(q_{2})}\left(\tau \right)\sinh\left(\theta_{q1}\right)\cosh\left(\theta_{q2}\right)\\ +{\tilde{f}}_{p(q_{2})}^{*}\left(\tau \right){\tilde{f}}_{u(q_{1})}\left(\tau \right)\sinh\left(\theta_{q1}\right)\sinh\left(\theta_{q2}\right)]R\left(f_{p}^{*},f_{u}\right)u_{q1}u_{q2}\\ +\frac{1}{N}\sum_{q_{1}}\sum_{Q}\sum_{p,u}^{M}A_{Qp}^{*}A_{Qu}\sinh^{2}\left(\theta_{q1}\right)R\left(f_{p}^{*},f_{u}\right)u_{q1}^{2}\\ +\frac{1}{N}\sum_{q_{1}q_{2}}e^{-i(q_{1}+q_{2})d}\sum_{Q}\sum_{p,u}^{M}A_{Qp}^{*}A_{Qu}[f_{p(q_{1})}^{*}\left(\tau \right)f_{p(q_{2})}^{*}\left(\tau \right)\cosh\left(\theta_{q1}\right)\cosh\left(\theta_{q2}\right)\\ +f_{p(q_{1})}^{*}\left(\tau \right){\tilde{f}}_{u(q_{2})}\left(\tau \right)\cosh\left(\theta_{q1}\right)\sinh\left(\theta_{q2}\right)+{\tilde{f}}_{u(q_{1})}\left(\tau \right){\tilde{f}}_{u(q_{2})}\left(\tau \right)\sinh\left(\theta_{q1}\right)\sinh\left(\theta_{q2}\right)\\ +f_{p(q_{2})}^{*}\left(\tau \right){\tilde{f}}_{u(q_{1})}\left(\tau \right)\sinh\left(\theta_{q1}\right)\cosh\left(\theta_{q2}\right)]R\left(f_{p}^{*},f_{u}\right)u_{q1}v_{q2}\\ +\frac{1}{N}\sum_{q_{1}}\sum_{Q}\sum_{p,u}^{M}A_{Qp}^{*}A_{Qu}\sinh\left(\theta_{q1}\right)\cosh\left(\theta_{q1}\right)R\left(f_{p}^{*},f_{u}\right)u_{q1}v_{q2}\\ +\frac{1}{N}\sum_{q_{1}q_{2}}e^{-i(-q_{1}-q_{2})d}\sum_{Q}\sum_{p,u}^{M}A_{Qp}^{*}A_{Qu}[f_{u(q_{1})}\left(\tau \right)f_{u(q_{2})}\left(\tau \right)\cosh\left(\theta_{q1}\right)\cosh\left(\theta_{q2}\right)\\ +{\tilde{f}}_{p(q_{2})}^{*}\left(\tau \right)f_{u(q_{1})}\left(\tau \right)\cosh\left(\theta_{q1}\right)\sinh\left(\theta_{q2}\right)+{\tilde{f}}_{p(q_{1})}^{*}\left(\tau \right)f_{u(q_{2})}\left(\tau \right)\sinh\left(\theta_{q1}\right)\cosh\left(\theta_{q2}\right)\\ +{\tilde{f}}_{p(q_{1})}^{*}\left(\tau \right){\tilde{f}}_{p(q_{2})}^{*}\left(\tau \right)\sinh\left(\theta_{q1}\right)\sinh\left(\theta_{q2}\right)]R\left(f_{p}^{*},f_{u}\right)v_{q1}u_{q2}\\ +\frac{1}{N}\sum_{q_{1}}\sum_{Q}\sum_{p,u}^{M}A_{Qp}^{*}A_{Qu}\cosh\left(\theta_{q1}\right)\sinh\left(\theta_{q1}\right)R\left(f_{p}^{*},f_{u}\right)v_{q1}u_{q2}\\ +\frac{1}{N}\sum_{q_{1}q_{2}}e^{-i(q_{2}-q_{1})d}\sum_{Q}\sum_{p,u}^{M}A_{Qp}^{*}A_{Qu}[f_{p(q_{2})}\left(\tau \right)f_{u(q_{1})}\left(\tau \right)\cosh\left(\theta_{q1}\right)\cosh\left(\theta_{q2}\right)\\ +f_{p(q_{2})}^{*}\left(\tau \right){\tilde{f}}_{p(q_{1})}^{*}\left(\tau \right)\cosh\left(\theta_{q2}\right)\sinh\left(\theta_{q1}\right)+{\tilde{f}}_{u(q_{1})}\left(\tau \right)f_{u(q_{1})}\left(\tau \right)\sinh\left(\theta_{q2}\right)\cosh\left(\theta_{q1}\right)\\ +{\tilde{f}}_{p(q_{1})}^{*}\left(\tau \right){\tilde{f}}_{u(q_{2})}\left(\tau \right)\sinh\left(\theta_{q1}\right)\sinh\left(\theta_{q2}\right)]R\left(f_{p}^{*},f_{u}\right)v_{q1}v_{q2}\\ +\frac{1}{N}\sum_{q_{1}}\sum_{Q}\sum_{p,u}^{M}A_{Qp}^{*}A_{Qu}\sinh\left(\theta_{q1}\right)\sinh\left(\theta_{q1}\right)R\left(f_{p}^{*},f_{u}\right)v_{q1}v_{q2}\\ +\sum_{Q}\sum_{p,u}^{M}A_{Qp}^{*}A_{Qu}R\left(f_{p}^{*},f_{u}\right)\sum_{q_{1}}v_{-q_{1}}^{2} \end{array}$$

Here $b_{d}^{†}$$\left(b_{d}\right)$ is the creation (annihilation) operator of the spin deviation at side d, which can be obtained from the magnon operators in the momentum space as follows [35]:

(A8) $$\begin{array}{c} b_{d}=N^{-1/2}\sum_{q}e^{i\mathrm{qd}}b_{q} \end{array}$$

(A9) $$\begin{array}{c} b_{q}=u_{q}\beta_{q}+v_{q}\beta_{-q}^{†} \end{array}$$
